# Manipulation of the Gut Microbiome Alters Acetaminophen Biodisposition in Mice

**DOI:** 10.1038/s41598-020-60982-8

**Published:** 2020-03-12

**Authors:** Michael A. Malfatti, Edward A. Kuhn, Deepa K. Murugesh, Melanie E. Mendez, Nicholas Hum, James B. Thissen, Crystal J. Jaing, Gabriela G. Loots

**Affiliations:** 1 Biosciences and Biotechnology Division, Lawrence Livermore National Laboratory, Livermore, CA 94550 USA; 2 School of Natural Sciences, University of California Merced, Merced, CA 95343 USA

**Keywords:** Pharmacokinetics, Microbiome

## Abstract

The gut microbiota is a vast and diverse microbial community that has co-evolved with its host to perform a variety of essential functions involved in the utilization of nutrients and the processing of xenobiotics. Shifts in the composition of gut microbiota can disturb the balance of organisms which can influence the biodisposition of orally administered drugs. To determine how changes in the gut microbiome can alter drug disposition, the pharmacokinetics (PK), and biodistribution of acetaminophen were assessed in C57Bl/6 mice after treatment with the antibiotics ciprofloxacin, amoxicillin, or a cocktail of ampicillin/neomycin. Altered PK, and excretion profiles of acetaminophen were observed in antibiotic exposed animals. Plasma C_max_ was significantly decreased in antibiotic treated animals suggesting decreased bioavailability. Urinary metabolite profiles revealed decreases in acetaminophen-sulfate metabolite levels in both the amoxicillin and ampicillin/neomycin treated animals. The ratio between urinary and fecal excretion was also altered in antibiotic treated animals. Analysis of gut microbe composition revealed that changes in microbe content in antibiotic treated animals was associated with changes in acetaminophen biodisposition. These results suggest that exposure to amoxicillin or ampicillin/neomycin can alter the biodisposition of acetaminophen and that these alterations could be due to changes in gut microbiome composition.

## Introduction

The effectiveness of drug treatments can vary wildly between individuals which can lead to decreased efficacy or increased adverse reactions. Much of the variation can be contributed to genetics, but environmental factors such as nutritional status, disease state, and gut bacterial composition can also profoundly influence the metabolic phenotype^1^. In recent years, the contribution of the gut microbiome on drug processing has been at the forefront of many studies investigating variations in drug response by the host. The gut microbiota is a vast and diverse microbial community residing in the human body that has co-evolved with its host to perform a variety of essential functions through a network of metabolism and signaling processes involved in the utilization of nutrients and the processing of xenobiotics^2^. Disruption of the microbiota whether induced by dietary changes, antibiotic administration or invasive pathogens can disturb the balance of the microbiota and alter metabolic networks. Such perturbations can affect the biodisposition of certain drugs, which can ultimately lead to adverse drug reactions. There are many diverse mechanisms the gut microbiome can use to alter the disposition, efficacy, and toxicity of drugs and xenobiotics^3^. These can include the expression of enzymes that can activate or inactivate drugs, the direct binding of drugs to a bacterial organism, the reactivation of drugs by microbial expressed enzymes and the direct competition between the host and microbes for host metabolizing enzymes. For example, an association between pre-dose, gut derived urinary metabolites and response to the commonly used analgesic acetaminophen has been reported^1^. Higher levels of predose urinary p-cresol sulphate predicted a reduction in the acetaminophen-sulphate: acetaminophen-glucuronide ratio post dose, indicating that gut derived microbial metabolites may increase acetaminophen toxicity by competing for the host enzyme sulfotransferase 1A1. Additionally, studies have shown that microbial derived reductive metabolism of digoxin can alter drug bioavailability leading to increased potentiation of the drug resulting in increased toxicity^4–6^. Although many studies have shown a relationship between gut microbiome composition and drug disposition, uncertainty still exists as to the specific interactions distinct microbial population might have on influencing drug biodisposition. Studies in germ-free and antibiotic treated rodents have shown that these conditions can affect the metabolic processing of acetaminophen. Germ-free mice have shown to have a greater sulfation capacity for acetaminophen conjugation^7^, while antibiotic treated rats have shown to have a diminished capacity to sulfate acetaminophen^8,9^. These conflicting results, and the lack of microbial characterization in these studies makes it difficult to ascertain the specific role the gut biome plays in acetaminophen metabolism. Microbes may also affect the rate at which drugs are absorbed from the gut. When orally administered probitics were given to mice the gut microbiome was altered, which caused changes in the pharmacokinetics of orally administered acetaminophen. Treatment with *Lactobacillus reuteri* caused a reduction of the acetaminophen area under the curve whereas treatment with *Lactobacillus rhamnosus* did not, indicating that gut microbial composition may affect the absorption of orally administered drugs^10^.

The current study lends further understanding to the contribution of the gut microbiota on acetaminophen biodisposition by investigating how specific changes in microbial composition can alter the metabolism and biodistribution of acetaminophen in C57Bl/6 mice. Treatment with the antibiotics ciprofloxacin, amoxicillin, or a cocktail of ampicillin/neomycin induced differential changes in gut microbe composition determined by the Lawrence Livermore Microbial Detection Array (LLMDA) and 16S rRNA sequencing. These particular antibiotics were chosen due to their broad-spectrum characteristics and different mechanisms of action which allow them to act on different bacterial phyla. Their relatively slow absorption rate allows for longer residence time in the gut after oral administration and have been shown to be affective at disrupting the gut microbiota^11–15^. Following exposure to carbon-14 labeled acetaminophen (^14^C-acetaminophen), altered plasma pharmacokinetics (PK), drug metabolism, and excretion profiles were observed in animals treated with antibiotics. The extent of observed changes in acetaminophen biodisposition were correlated with the changes in microbiome composition due to individual antibiotic treatments. The results suggest that disruption of specific gut bacterial composition can differentially alter the bioavailability of acetaminophen and that these alterations could potentially affect drug efficacy.

## Results

### Plasma concentration of acetaminophen

**Figure 1:**
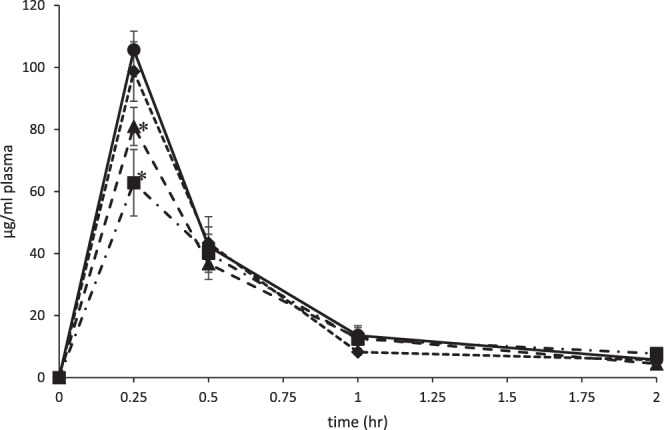
Plasma concentration time profiles of acetaminophen. Plasma concentration time profiles of a single oral dose acetaminophen (100 mg/kg) following a 10-day oral exposure to antibiotics in male C57Bl/6 mice. Control (•), ciprofloxacin (♦), amoxicillin (▲), ampicillin/neomycin (■). Data is expressed as the mean (n = 4) ± SE. *p < 0.05.

The plasma concentration of acetaminophen was obtained after administration of a single 100 mg/kg oral dose of ^14^C-acetaminophen, following either a 10-day exposure to antibiotics through drinking water or control water without antibiotic. Mean plasma concentrations of acetaminophen (based on total radioactivity) over time are illustrated in Fig. 1. The mean pharmacokinetic parameters are presented in Table 1. The plasma concentration time curves for all exposure groups were similar with the T_max_ occurring at the first measured time point of 0.25 h. There was a statistically significant (*p* < 0.05) decrease in the C_max_ of acetaminophen in the amoxicillin and ampicillin/neomycin treated groups compared to the control antibiotic-free group. The mean acetaminophen C_max_ for animals receiving amoxicillin or ampicillin/neomycin was 80.9 µg/ml plasma and 62.8 mg/ml plasma, respectively, whereas the C_max_ for the control group was higher at 105.6 µg/ml plasma. These reductions in C_max_ suggest reduced bioavailability in the amoxicillin and ampicillin/neomycin treated groups compared to controls. There was no observed significant difference in C_max_ in the ciprofloxacin treated group compared to controls. The mean half-life (T_1/2_) for the ampicillin/neomycin exposed animals was 0.66 hr which was 1.4X longer compared to the control animals (0.46 hr). There was no change in the T_1/2_ from the amoxicillin or ciprofloxacin treated groups (Table 1).

**Table 1:** Pharmacokinetic parameters of acetaminophen from a single oral administration of 100 mg/kg ^14^C-acetaminophen following exposure to antibiotics in C57Bl/6 mice.

Treatment	C_max_ (µg/mL)	T_max_ (hr)	T_1/2 (hr)_	AUC (µg•hr/mL)
control	105.6	0.25	0.46	55.3
ciprofloxacin	98.6	0.25	0.48	49.9
amoxicillin	80.9*	0.25	0.48	45.8
ampicillin/neomycin	62.8*	0.25	0.66	43.9

*Significantly different from controls. (*p* < 0.05).

### Tissue distribution of acetaminophen

^14^C-Acetaminophen was detected in all tissues examined from all treatment groups with the stomach having the highest levels averaging between 0.4–1.3 nmol/mg tissue, followed by the kidney, intestine, and liver. In the amoxicillin and ampicillin/neomycin treated animals, higher levels of acetaminophen were measured in the stomach and intestine compared to controls, although these observations were not statistically significant (Fig. S1).

### Acetaminophen metabolism

Chromatographic analysis of the urine samples revealed 2 major radioactive peaks accounting for approximately 90% of the detected radiolabel. In the control animals, the first peak eluting at 5 minutes accounted for 10.84% of the radiolabel and corresponded to an acetaminophen-sulfate conjugate; a peak eluding at 20 minutes accounted for 78.87% of the radiolabel and was identified as the acetaminophen-glucuronide conjugate (Table 2). Metabolite identification was based on susceptibility to sulfatase and β-glucuronidase. All other metabolites were relatively minor and were not characterized. In both the amoxicillin and the ampicillin/neomycin treated animals, the sulfate metabolite was decreased to 7.49% and 2.71% of the recovered radioactivity respectively, relative to controls, with the ampicillin/neomycin samples having a statistically significant decrease (*p* ≤ 0.05) relative to controls. (Table 2). There was also a concomitant increase in the glucuronide metabolite. Interestingly, in the ciprofloxacin treated samples the opposite trend was observed, with the sulfate metabolite increasing in concentration to 15.11% and the glucuronide metabolite decreasing to 67.52%. These changes were reflected in the sulfate-to glucuronide ratios (Table 2). In the control samples the sulfate-to-glucuronide ratio was 0.14, whereas for the amoxicillin and ampicillin/neomycin treated samples it was 0.09, and 0.03, respectively, and for the ciprofloxacin treated samples it was 0.22. Fecal metabolites were not analyzed since urinary excretion is the major route of elimination for acetaminophen^16^.

**Table 2:** Acetaminophen metabolites recovered in mouse urine after a single oral administration of 100 mg/kg ^14^C-acetaminophen following exposure to antibiotics in C57Bl/6 mice.

Treatment group	Sulfate	Glucuronide	Sulfate/Glucuronide
Control	10.84 ± 2.85	78.87 ± 2.99	0.14
Ciprofloxacin	15.11 ± 2.51	67.52 ± 5.19*	0.22*
Amoxicillin	7.49 ± 1.34	79.91 ± 1.39	0.09
Ampicillin/neomycin	2.71 ± 0.26*	87.54 ± 1.27*	0.03*

Data is expressed as the mean percent recovered radioactivity (n = 4 ± SD).

*Significantly different from controls. (*p* < 0.05).

### Recovery of urinary and fecal radioactivity

Clearance of acetaminophen occurred primarily through the renal elimination route with the majority of the recovered radiolabel being excreted in the urine. In control and ciprofloxacin treated animals over 93% of the radiolabel was excreted in urine with less than 7% in the feces (Table 3). However, in amoxicillin and ampicillin/neomycin treated animals the amount excreted in the urine was reduced to 87.5% and 87.4%, respectively, with concomitant increases in the amount recovered in the feces. The greatest change in the urine to feces excretion ratio was from the ampicillin/neomycin treated animals followed by the amoxicillin treated group. This trend is similar to the observations reported in the plasma showing the greatest change in plasma C_max_ occurring in the ampicillin/neomycin group followed by amoxicillin treated animals compared to the control and ciprofloxacin treated groups.

**Table 3:** Urinary and fecal excretion of total radioactivity over 24 hr. Expressed as percent of recovered ^14^C radiolabel.

excretion route	control	Ciprofloxacin	Amoxicillin	Ampicillin/neomycin
urine	93.3 ± 1.4	95.7 ± 1.3	87.5 ± 5.1	87.4 ± 1.0*
feces	6.7 ± 1.4	4.3 ± 1.3	12.5 ± 5.1	12.6 ± 1.0*

Data are expressed as the percent of recovered 14 C radiolabel ± SD from 4 mice per treatment group.

*Change in urine and fecal recovery of radioactivity statistically different from control. (*p* < 0.05).

### Microbial characterization

**Figure 2:**
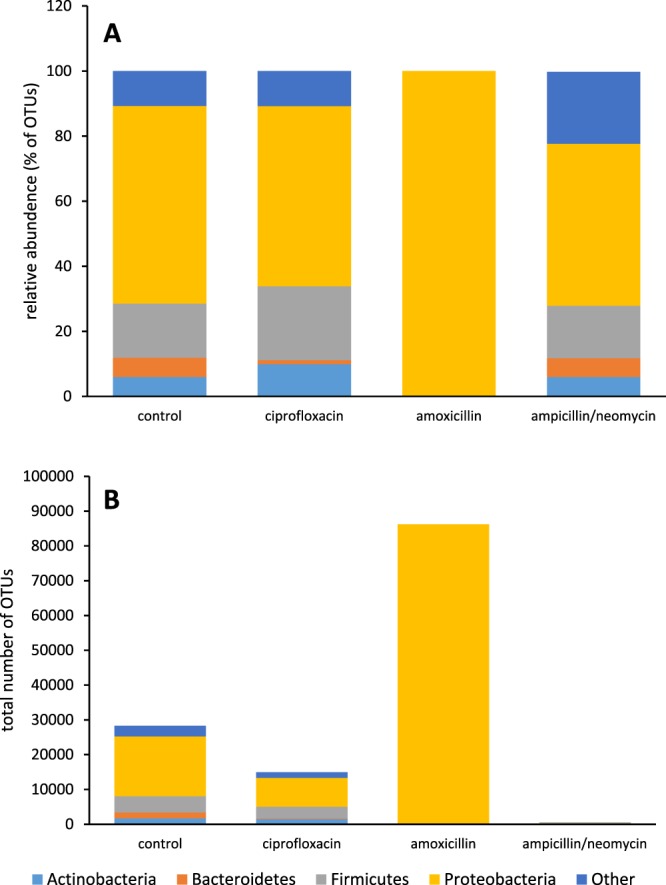
16S rRNA sequencing analysis of isolated fecal DNA. 16S rRNA sequencing analysis of fecal DNA following a 10-day oral exposure to ciprofloxacin, amoxicillin, or ampicillin/neomycin antibiotics in male C57Bl/6 mice. (**A**) relative abundance of all detected phyla in each treatment group. Data is expressed as percent of total OTUs. (**B**) Total number of OTUs per detected phyla in each treatment group.

A qualitative representation of the microbial species detected in fecal samples derived from each treatment group by LLMDA are shown in Table 4. The analysis revealed significant decreases in microbial diversity in both the amoxicillin and ampicillin/neomycin treated groups compared to the no antibiotic group. In the control group *Firmicutes Actinobacteria, Bacteroidetes* and *Proteobacteria* were the dominant phyla. In the amoxicillin treated group only *Proteobacteria* and a *Trypanosomatidae* were detected. Four *Proteobacterial* families not detected in the control group were observed in the amoxicillin treated group. These included the gram negative, *Alcaligenaceae, Burkholderiaceae, Piscirickettsiaceae*, and *Succinivibrionaceae* families. In the ampicillin/neomycin treated group, no microbes were detected above the threshold limit set for the assay. Treatment with ciprofloxacin had the least effect on microbial diversity with 14 microbial families detected; 11 overlapping with controls, and 3 not seen in the control group (Table 4). 16S rRNA sequencing of the isolated fecal DNA revealed similar compositional makeup of phyla between the controls, the ciprofloxacin treated groups, and the ampicillin//neomycin treated group with *Proteobacteria* being the dominant phyla followed by *Firmicutes* and *Actinobacteria* (Fig. 2A). A significant decrease in bacterial diversity was observed in the amoxicillin treated group with 100% of the operational taxonomic units (OTUs) mapping to *Proteobacteria*. This decrease in diversity was accompanied by an increase in the total number of *Proteobacteria* OTUs compared to controls. OTUs are defined as sequence reads clustered into bins based on a similarity threshold of 97%. When total OTUs are considered, there was a 47.2% and 98.4% reduction in total OTUs in the ciprofloxacin and ampicillin/neomycin treated groups, respectively (Fig. 2B). In the amoxicillin group a 204.3% increase in total OTUs was observed.

**Table 4:** Microorganisms detected by LLMDA from microbial DNA isolated from mouse fecal samples.

Phyla	Family	10-day water only	10-day Cipro 125 mg/L	10-day amox 950 mg/L	10-day amp/neo 1.0 g/L
Actinobacteria	*Bifidobacteriaceae*	x			
	*Coriobacteriaceae*	x			
	*Intrasporangiaceae*	x	x		
	*Pseudonocardiaceae*	x			
Bacteroidetes	*Marinilabiliaceae*	x			
	*Prevotellaceae*	x	x		
	*Rikenellaceae*	x			
Euglenozoa	*Trypanosomatidae*			x	
Firmicutes	*Carnobacteriaceae*	x			
	*Clostridiaceae*	x	x		
	*Clostridiales*	x	x		
	*Erysipelotrichaceae*	x	x		
	*Eubacteriaceae*	x	x		
	*Lachnospiraceae*	x	x		
	*Lactobacillaceae*	x	x		
	*Peptococcaceae*	x	x		
	*Ruminococcaceae*	x			
Proteobacteria	*Alcaligenaceae*			x	
	*Burkholderiaceae*			x	
	*Burkholderiales*		x		
	*Desulfohalobiaceae*	x	x		
	*Gammaproteobacteria*	x	x	x	
	*Legionellaceae*	x			
	*Neisseriaceae*		x		
	*Piscirickettsiaceae*			x	
	*Succinivibrionaceae*			x	
Spirochetes	*Spirochaetaceae*	x			
Synergistetes	*Synergistaceae*	x			
Tenericutes	*Mycoplasmataceae*	x			
Verrucomicrobia	*Verrucomicrobiaceae*	x	x

## Discussion

The effects the gut microbiome has on human health are well documented^17^. In addition to their ability to perform numerous important biochemical functions for the host, they are also capable of performing a range of biotransformations on xenobiotics, such as drugs and their metabolites in ways that can affect absorption and bioavailability. The current study investigated how manipulation of the gut microbiome through treatment with antibiotics can alter the biodisposition of the analgesic acetaminophen. This in the first study to show how specific changes in gut microbiome diversity and abundance, using microarray and 16SrRNA sequencing, can affect acetaminophen biodisposition. Results show that exposure to amoxicillin or a cocktail of ampicillin and neomycin daily for 10 days prior to an oral exposure to acetaminophen caused a decrease in the plasma concentration of acetaminophen. This was evidenced by a significant decrease in the plasma C_max_. A small non-statistically significant decrease in AUC_0−t_ was also observed. This lack of change in AUC is not uncommon for altered absorption kinetics for acetaminophen^18^. These changes reflect a decrease in whole body exposure and bioavailability of the drug resulting in the possibility of reduced efficacy. These observations are in contrast to a previous study that reported an increase in both plasma C_max_ and AUC in pseudo germ-free rats orally exposed to a 200 mg/kg dose of acetaminophen compared to control conventional animals^8^. The pseudo germ-free status was obtained by orally administering an antibiotic cocktail consisting of bacitracin, streptomycin and neomycin twice daily for 5 days. The differences in C_max_ and AUC between the studies could be attributed to differences in acetaminophen dose administered, the difference in antibiotic exposure time, or the distinctive changes in the microbial composition of the gut, specific to the different antibiotic treatments, or to the antibiotics themselves.

Studies have shown that the rate of drug absorption from the gut into the systemic circulation can be influenced by the diversity and abundance of gut microbes. Matuskova *et al*. has reported that the oral administration of *E.coli* strain Nissle 1917 to rats increased the absorption of the drug amiodarone from the gastrointestinal tract^19^. It has also been reported that several bacterial membrane proteins found in gut microbes can function as drug transporters facilitating transport of drugs across the gastrointestinal tract^20^. Therefore, it can be inferred that the absence of these bacteria and bacterial transporters from the gut biome can decrease the absorption of drugs into the circulation. Most studies have shown, however, that absorption of acetaminophen into the systemic circulation occurs primarily through passive diffusion and that the greatest amount of absorption occurs in the proximal portion of the small intestine with very little being absorbed from the stomach. Thus, the rate limiting step in acetaminophen absorption is gastric emptying (reviewed 18). The higher levels (although not statistically significant) of acetaminophen in the stomach from the amoxicillin and ampicillin/neomycin treated animals compared to controls suggests that treatment with these antibiotics can possibly slow gastric emptying time limiting absorption into the systemic circulation. The decrease in the elimination rate constant (k_el_) for the stomach from the antibiotic treated animals re-enforces this concept. The elimination rate constant was determined by obtaining the slope from the linear regression derived from the semi-log plot of the concentration *vs* time data. The slope of the line is equal to −k_el_. It would also be expected that a slower gastric emptying time would delay the plasma T_max_. This, however, was not observed in the current study. Although, the mean plasma T_1/2_ for the ampicillin/neomycin treated animals was 1.4x longer compared to the control animals. The lack of change in T_max_ could be attributed to the observed higher levels of acetaminophen in the stomach, after antibiotic treatment, not being significant enough to change the T_max_ to any great extent. Furthermore, since the plasma sampling interval was 15 min (15–30 min) it is possible that a small delay in T_max_ would not have been captured with the current sampling interval. Therefore, to consider these factors, the decrease in bioavailability, due to alterations in the gut microbiome from antibiotic treatment may not be due to a decrease in absorption per se but to a decrease in gastric emptying, therefore, limiting the amount of drug entering the systemic circulation.

Antibiotic treatment also affected acetaminophen metabolism, as shown by the decrease in the acetaminophen-sulfate conjugate in the urine of amoxicillin and ampicillin/neomycin treated animals. Similar results were reported in previous studies showing a decrease in the ratio of the AUC of the acetaminophen-sulfate conjugate in the plasma of rats orally administered bacitracin, streptomycin and neomycin prior to an oral acetaminophen exposure^8^, and in rats treated with a cocktail of chloramphenicol, nystatin, streptomycin, erythromycin and penicillin followed by acetaminophen exposure, a 15% decrease in the acetaminophen sulfate conjugate was observed compared to controls^9^. Conversely, studies in germ-free mice have reported increases in the sulfate conjugate in the urine of acetaminophen treated mice compared to conventional controls^7^. These differential effects could have resulted from many factors including variation in the expression of metabolizing enzymes in the host animal and/or competitive inhibition of enzyme capacity by microbial-derived compounds in animals with a full complement of intestinal bacteria. Changes in drug metabolizing capabilities in both the gut and liver, due to differential expression of bacterial populations, have been reported^21^. Results obtained from comparing liver preparations from germ-free and conventional rats revealed differences in the expression of cytochrome P450s capable of bioactivating mutagenic heterocyclic amines^22^. Some of these same cytochrome P450s are involved in the metabolism of acetaminophen^23^. Although the possibility of bacteria contributing to acetaminophen metabolism cannot be dismissed^10^, there is no evidence that acetaminophen itself is metabolized to any great extent by the gut microbiota^21,24^. One study did show that the administration of the probiotic *Lactobacillus reuteri* to mice caused a degradation of acetaminophen when incubated with a mouse fecal suspension. This degradation was attributed to the probiotics affecting the rate of absorption of acetaminophen by altering the composition of the gut microbiota^10^. No evidence was presented to support the direct metabolism of acetaminophen by the gut microbiota.

Another factor to consider is the hydrolysis of the conjugated metabolites by microbial enzymes affecting urinary metabolite levels. A major effect of these enzymes is the hydrolysis of biliary-excreted conjugated metabolites (such as glycine, glucuronide and sulfate conjugates) and the re-establishment of the aglycone which enables reabsorption of the drug *via* enterohepatic recirculation^21^. It has been established that β-glucuronidases can deconjugate the acetaminophen-glucuronide allowing for reabsorption of the parent drug, at which point, it can be further metabolized and re-conjugated as the sulfate and/or glucuronide conjugate. β-Glucuronidases are widely distributed across many gut bacterial species including members of the Proteobacteria, Firmicute, and Actinobacteria phyla^25^ providing for efficient deconjugation of acetaminophen-glucuronide in animals with a normal population of gut bacteria. Therefore, of consideration in the current study, the decrease in the acetaminophen-sulfate metabolite in the urine of the amoxicillin and ampicillin/neomycin treated animals could be due to a lack of bacterial glucuronidase activity due to decreased bacterial populations, as well as increased biliary excretion. Therefore, the amount of reabsorption of the parent acetaminophen would be limited, consequently diminishing the potential of further sulfate conjugation as evidenced by the decrease in urinary acetaminophen-sulfate metabolite in the antibiotic treated animals. A decrease in acetaminophen sulfate was also observed in rats pre-treated with an antibiotic cocktail of chloramphenicol, nystatin, streptomycin, erythromycin and penicillin^9^. In the control animals it would be expected that reabsorption of acetaminophen would be increased, evidenced by an increase in the plasma concentration at later time points. This, however, was not observed when plasma was monitored out to 24 hr (data not shown). There was no detectable ^14^C in any of the plasma samples post 2 hr after dosing.

A leading question with the current results is: are the observed effects on acetaminophen biodisposition due to the exposure to antibiotics or to the changes in gut biome composition as a consequence of antibiotic exposure? The LLMDA results together with the 16S rRNA analysis showed a differential response to the different antibiotic treatments and the differential response observed in acetaminophen plasma concentration and urinary metabolism profiles from the 3 antibiotic treatment groups suggests that the effects are due to changes in bacterial composition and not to antibiotic exposure. The LLMDA assay allowed for a survey of fecal bacteria diversity and 16S rRNA sequencing provided the relative abundance of each phyla present in the gut biome before and after antibiotic treatment.

The microbial diversity was relatively unchanged between the control and ciprofloxacin treated groups with *Proteobacteria* and *Firmicutes* constituting of the largest proportions of bacteria, whereas in the amoxicillin treated groups, the diversity was significantly reduced with *Proteobacteria* dominating post treatment. Overall bacterial abundance, measured as the total number of OTUs, was affected by antibiotic treatment with the ciprofloxacin treated group showing a slight decrease in abundance and the ampicillin/neomycin group having the greatest effect, depleting nearly all of the detectable phyla. The ciprofloxacin finding is to be expected due to its limited effect against anerobic bacteria^26^. These results corresponded with the results showing ciprofloxacin treatment having no observable effect on acetaminophen plasma concentrations or metabolism and ampicillin/neomycin treatment having the greatest effect. Interestingly, in the amoxicillin group the total number of *Proteobacteria* OTUs actually increased compared to controls suggesting that the reduction in bacterial diversity stimulated the growth of opportunistic *Proteobacteria* presumably due to less competition for resources. An increase in *Proteobacteria* OTUs was also reported in a similar study in rats treated with amoxicillin. 16S rRNA sequencing revealed a significant decrease in bacterial diversity coupled with an with an increase in *Protobacteria* density and a reduction in *Firmicutes* compared to untreated controls^27^. Additionally, in a study investigating the effect of antibiotics on the human gut microbiome, sequence analysis from humans treated with amoxicillin-clavulanate showed significantly decreased microbial diversity while increasing the microbial load of gram negative bacteria by 2-fold^28^.

These results reinforce the concept that the composition of the gut microbiome is an essential consideration in determining the metabolic response of the host to xenobiotics. Manipulation of the gut microbiome can have consequences for drug disposition. Therefore, for optimal drug efficacy, one’s microbial composition should be considered when determining the response to pharmacological therapies.

## Materials and Methods

### Chemicals

^14^C-acetamenophen (specific activity, 80 mCi/mmol) was purchased from American Radiolabeled Chemicals, Inc. (St Louis, MO). Radio-chemical purity was assessed by HPLC and determined to be >98% pure. Unlabeled acetaminophen, ciprofloxacin, ampicillin, and neomycin were purchased from Sigma –Aldrich (St. Louis, MO). Amoxicillin was obtained from Virbac AH Inc. (Fort Worth, TX).

### Animal study design

Animal experiments were conducted at the Lawrence Livermore National Laboratory (LLNL) AAALAC accredited animal care facility. The animal study protocol was reviewed and approved by the LLNL Institutional Animal Care and Use Committee (IACUC) prior to the initiation of the study. All experiments were performed in accordance with relevant guidelines and regulations set forth by the Guide for the Care and Use of Laboratory Animals (Eighth Ed. 2011). Male C57Bl/6 mice weighing 25–30 g were obtained from The Jackson Laboratory (Bar Harbor, Me). Mice were housed in groups of four in polystyrene cages containing corncob bedding and kept on a 12 h light/dark cycle in a ventilated room maintained at 24 °C. Food and water were provided *ad libitum*. Mice were randomly divided into 4 treatment groups according to Table S1. Each mouse was exposed to either ciprofloxacin (125 mg/L)^13^, amoxicillin (950 mg/L)^14^ or an ampicillin/neomycin cocktail (1 g/L:0.5 g/L)^15^ in their drinking water for 10 days, or sterile tap water as a control. Antibiotic solutions were renewed every 3^rd^ day. On day 10 mice were treated with a single oral gavage dose of 100 mg/kg ^14^C-acetaminophen (specific activity, 0.37 µCi/mmol). Following ^14^C-acetaminophen administration animals were euthanized by CO_2_ asphyxiation at the time points designated in Table S1, and blood and tissues collected. Within one hour of collection the plasma was separated from the whole blood by centrifugation (8,000 × g for 2 min). The volume of plasma obtained was recorded and the samples were stored at −80 °C until analysis by accelerator mass spectrometry (AMS). Tissues (liver, kidney, stomach, intestine) were excised from the carcass, rinsed in PBS and subsequently placed in clean 20 ml volume glass vials and stored at −20 °C until analysis by liquid scintillation counting (Packard, Perkin Elmer, Waltham, MA). Fecal pellets were collected before antibiotic exposure and prior to acetaminophen dosing for isolation of bacterial DNA present in the pellets to determine gut microbe composition, before and after antibiotic treatment. A subset of animals was placed in metabolism cages and urine and feces were collected for 24 h after ^14^C-acetaminophen administration for metabolite analysis.

### Plasma concentration over time

The concentration of acetaminophen in plasma was determined by quantifying the amount of ^14^C equivalents in plasma, using AMS, at time points up to and including 2 h and constructing concentration vs. time curves^29^. A two-stage approach was used to independently fit the plasma concentration data from each mouse, and then determine the means ± standard errors. The maximal concentration observed in plasma (C_max_) was determined from the concentration-versus-time data. The area under the curve (AUC) was calculated for the intervals from time zero to time t (AUC_0−t_), where t is the time of the last measured concentration, using the linear trapezoidal method.

### Tissue distribution of acetaminophen

The concentration of acetaminophen in tissue was assessed by quantifying the levels of C-14 in each collected tissue after tissue homogenization and digestion, with subsequent analysis by liquid scintillation counting (LSC). A 100–200 mg portion of each tissue was rinsed with phosphate buffered saline until no radioactivity could be detected in the wash buffer. Tissues were subsequently solubilized by adding 1–2 ml of Soluene-350 tissue solubilizer (PerkinElmer Inc., Waltham, MA) and incubated in a 60 ° C water bath with occasional vortexing for 1–4 hr, to ensure complete solvation of the tissue. After incubation the samples were allowed to cool to room temperature and then 0.2 ml of 30% hydrogen peroxide was added in two 0.1 ml aliquots with swirling between additions. Samples were then heated at 60 ° C for 1 hr to complete the decolorization process. For analysis of feces, approximately 100 mg of fecal material was added to 1.0 mL sodium hypochlorite. The samples were vortexed mixed and then incubated in a water bath at 60 ° C for 1 hr with occasional vortexing. After the 1 hr incubation an additional 1.0 ml of sodium hypochlorite was added and the samples were incubated an additional 1–2 hr until complete digestion. All samples were allowed to cool to room temperature. Finally, 10 mL of Hionic-Fluor scintillation cocktail (PerkinElmer Inc., Waltham, MA) was added to each sample and placed in the dark for 24 hr prior to counting by LSC. (PerkinElmer, Packard).

### Acetaminophen metabolism

To assess the extent of acetaminophen metabolism, urine was collected over 24 h post exposure of the ^14^C-acetaminophen dose and immediately frozen at −20 °C. Prior to HPLC analysis, each urine sample was thawed, and a 0.1–0.5 mL aliquot from each fraction was analyzed by liquid scintillation counting to determine the total 14-carbon content. Each sample was then analyzed by reversed-phase HPLC for acetaminophen and acetaminophen metabolites. After centrifugation of approximately 150 µL of each urine sample at 10,000 RPM for 5 min, 100 µl of the supernatant was directly injected into an Alliance HPLC system (Waters, Milford, MA) equipped with a 4 μm, 4.6 × 250 mm Synergi Max-RP 80 A column (Phenomenex, Torrance, CA), and monitored at 248 nm. Metabolites were eluted at 1.0 ml/min using a solvent of 88% 0.1 M KH_2_PO_4_ containing 0.75% glacial acetic acid and 12% methanol. The column eluent was collected at 1 min intervals, and radioactivity was quantified by liquid scintillation counting.

Acetaminophen glucuronide and sulfate conjugates were determined based on their susceptibility to β-glucuronidase and sulfatase, respectively^30^.

### Microbial detection array

Microbial diversity in the collected fecal samples was assessed using the Lawrence Livermore Microbial Detection Array (LLMDA). The LLMDA is the most comprehensive microorganism detection platform built to date and the first high throughput microarray that contains DNA probes capable of whole genome resolution for identifying all sequenced microbes^31^. This array can detect any sequenced viruses or bacteria within 24 h. It has been used to detect a wide range of both bacteria and viruses^32,33^ and has been validated to probe for all known microbiological agents for which whole genomes are available. Proof of concept has been demonstrated by the application of microarrays for use in clinical medicine, food safety testing, environmental monitoring, and for biodefense^34–40^. Fecal samples were collected from individually housed antibiotic treated and control mice and stored at −20 °C until DNA extraction. For extraction of DNA, 0.25 g of fecal material from each sample was extracted using the QIAmp PowerFecal DNA kit (Qiagen, #12830–50) following standard manufacturer procedures. DNA samples were resuspended in Buffer C6 and quantitated using the Qubit fluorometer (ThermoFisher Scientific). LLMDA processing utilized the version (v7 4 × 180 K) of the LLMDA manufactured by Agilent Technologies and contains probes designed to detect the following number of microbial species: 3,856 viruses, 3,855 bacteria, 254 archaebacteria, 100 fungi, and 36 protozoa^41^. Following DNA extraction, 750 ng of each sample was fluorescently labeled using Cy3 5′ labeled random nonamer primers as described previously^33,41–43^. After labeling, the samples were loaded onto the LLMDA and hybridized at 65 °C for 40 hours in a rotating oven. At the end of 40 hours, the microarrays were washed using standard manufacturer procedures with CGH wash buffers (Agilent, #5188–5226). Microarrays were scanned utilizing a Roche MS200 microarray scanner at a resolution of 2 µm. The intensity of each feature on the array was calculated and extracted using the Feature Extraction Software (Agilent). The extracted data was then analyzed using the CLiMax method in which the log likelihood of each of the potential microbial targets is estimated from the BLAST similarity scores of the array feature and target sequences, together with the feature sequence complexity and other covariates derived from the BLAST results, as described previously^31^.

### 16S rRNA sequence analysis

To determine microbial relative abundance in the collected fecal samples 16S rRNA sequencing was utilized^44–47^. DNA was extracted from fecal samples as described above. The resulting DNA was suspended in TE buffer. 16S rRNA gene sequences, corresponding to the hypervariable V3-V4 region were generated from each isolated DNA in separate PCR-reactions using the Illumina MiSeq sequencing platform (Illumina, San Diego, CA). Primers used were adapted from Klindworth *et al*.^48^ with Illumina adapter overhang sequences added to the gene-specific sequence: V3F: 5′-F:TCGTCGGCAGCGTCAGATGTGTATAAGAGACAGCCTACGGGNGGCWGCAG-3′;V4R: 5′-GTCTCGTGGGCTCGGAGATGTGTATAAGAGACAGGACTACHVGGGTATCTAATCC-3′. Dual index codes were added to each sample using Nextera XT index kit v2 Set B (Illumina) prior to sequencing. Metagenomic analysis of amplified regions were performed using MiSeq reporter (Illumina) to classify bacterial identities.

### Statistics

All values are expressed as the mean of 4 replicates per treatment group ± the standard deviation. All comparisons were done using the Students *t*-test. Comparisons with *p*-values < 0.05 were considered statistically significant.

## Supplementary information

Supplementary information.

## Data Availability

The datasets generated during and/or analyzed during the current study are available from the corresponding author on reasonable request.
