# Supplementary material for: Manipulation of the Gut Microbiome Alters Acetaminophen Biodisposition in Mice
Source: Sci Rep. 2020 Mar 12;10:4571. doi: 10.1038/s41598-020-60982-8 (PMC7067795; doi:10.1038/s41598-020-60982-8)
Supplement: Supplementary file 1 — Supplementary information. [file 41598_2020_60982_MOESM1_ESM.pdf]

Supplementary Information:

Manipulation of the Gut Microbiome Alters Acetaminophen Biodisposition in Mice.

Michael A. Malfatti, Edward A. Kuhn, Deepa K. Murugesh, Melanie E. Mendez, Nicholas Hum, James B. Thissen, Crystal J. Jaing, Gabriela G. Loots

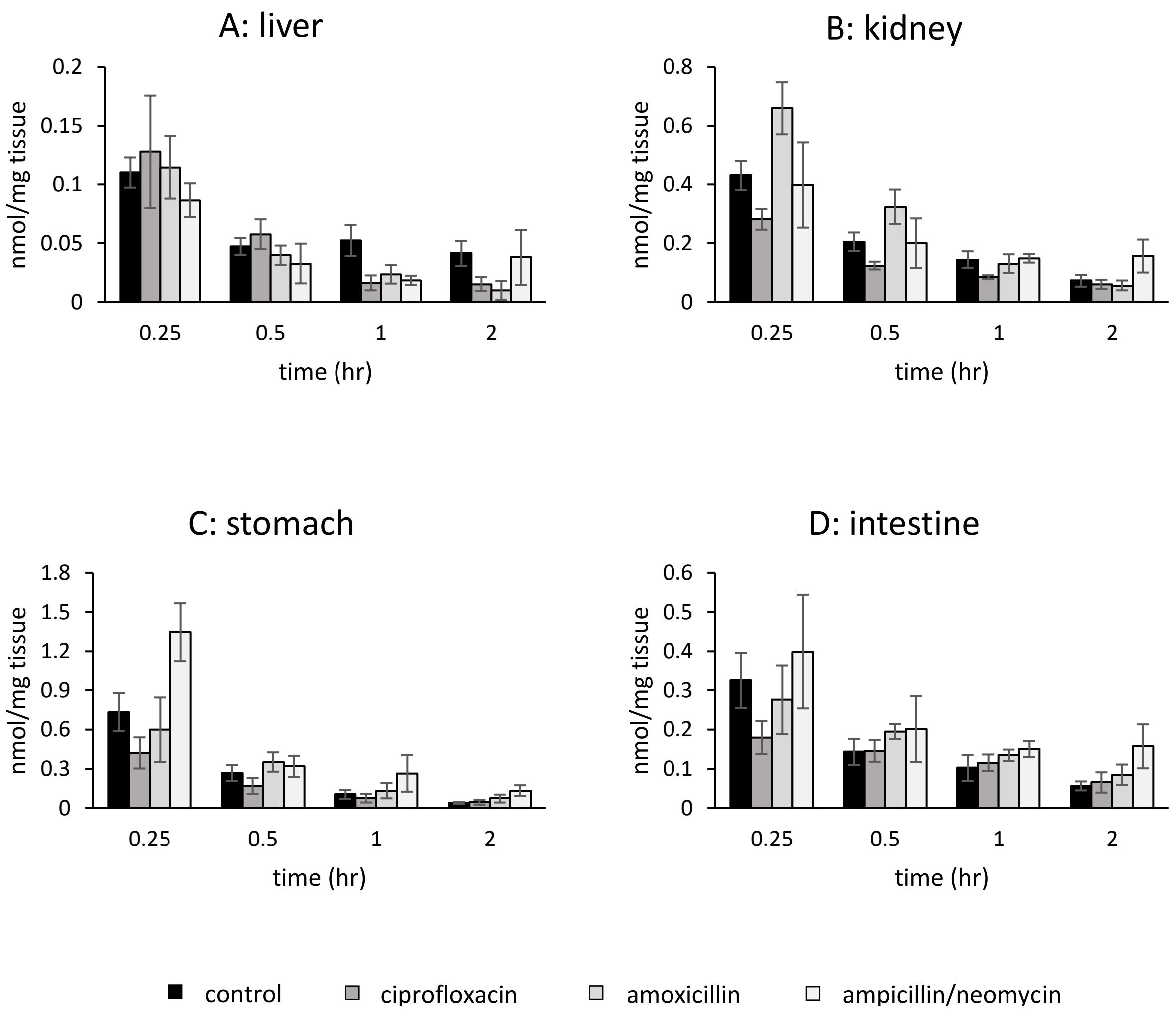

Figure S1. **Tissue distribution of acetaminophen**. Mean tissue concentration of a single oral dose acetaminophen (100 mg/kg) following a 10-day oral exposure to antibiotics in male C57Bl/6 mice. Data is expressed as the mean (n=4) ± SE.

Table S1. Experimental Study design

Group	Treatment	Sample collection time (hr)	Tissues collected
1, n=4/time pt.	^{14}C-acetaminophen/water	0, 0.25, 0.5, 1, 2	blood, liver, kidney, stomach, intestine
2, n=4/time pt.	^{14}C-acetaminophen/ciprofloxacin	0, 0.25, 0.5, 1, 2	blood, liver, kidney, stomach, intestine
3, n=4/time pt.	^{14}C-acetaminophen/amoxicillin	0, 0.25, 0.5, 1, 2	blood, liver, kidney, stomach, intestine
4, n=4/time pt.	^{14}C-acetaminophen/ampicillin/ neomycin	0, 0.25, 0.5, 1, 2	blood, liver, kidney, stomach, intestine
